# Dentistry and Otopathy

**Published:** 1884-03

**Authors:** F. Park Lewis

**Affiliations:** Buffalo, N. Y.


ARTICLE II.

### DENTISTRY AND OTOPATHY.

BY F. PARK LEWIS, M. D., BUFFALO, N. Y.

(Read before the 7th and 8th District Dental Societies of New York, October, 1883.)

There is no fact in the domain of science that can be isolated from all other facts. There is no force in nature that could exist were it not for other and perhaps antagonistic forces. As we endeavor to penetrate the infinite depths of space, we realize that far beyond the distant worlds that twinkle as fireflies are other worlds grander in their magnitude than is ours; that beyond these is star dust which our telescopes resolve into the marvelous mystery of new systems, with their own suns and moons, and in the immeasurable beyond is still star dust which we cannot resolve. But by a power immutable and unvarying the far-away alcyone is bound by indissoluble bonds to the tiniest grain of sand upon the seashore. The little dancing wavelets leaping in the exuberance of an imagined freedom are but the messengers of the moon, and go and come as she may bid them; and even that solitary sentinel completes her vigils as our own earth directs. Among all the nations of men, there is not one that does not bear the imprint of its neighbor's qualities. In custom, language and thought the fact of mutual relationship predicates an influence that will modify and change—a catalysis, to speak with the chemist, that cannot be prevented.

There is, in a word, in the universe of nature a mutual dependence of power on power, and force on force, by which the elision of the smallest factor is precluded in the attainment of a given result. The introduction of a single tone will render discordant the general harmony. It is by the contingent action of each on each that the balance is maintained.

In the human economy this is none the less a fact. There is no organ, no tissue, in which a functional disturbance may not interfere with the general integrity; and conversely a depreciation of the general tone may be chiefly exhibited in a single structure in which the intensity of morbific action has been centered. In the study of disease the application of more scientific principles and the development of each of the branches of medical and surgical practice have compelled our most faithful workers to limit their labors by arbitrary lines. It is to the unselfish labors of these men that medicine to-day owes the proud position that it now occupies, and if their ultimate reward is not the attainment of sought-for truth, they are at least the daily gleaners of collateral facts that unite to form the aggregate of attainable knowledge. Whether the discoveries of Pasteur and of Bastian and of Koch shall prove to be etiological verities or not, medical science and humanity rest under obligations to all these men for having substituted observation for speculation; and whether the facts observed can be harmonized with the deductions which have been drawn from them or not, the *facts* still remain as bases for future investigation. In their patient and wearisome labors in the field of pathology, Virchow and Pager and Rokatansky have given to the world a knowledge of disease that has anchored the shifting hypotheses upon the solid bases of verified knowledge.

Surgeons had tentatively and hesitatingly explored the *erra incognito* of ophthalmology before the days of Desmarres and Jæger and Arit, but it was only when these men had begun to study the physiological phenomena which the human eye disclosed, and when their brilliant pupil Von Graafe had applied the ophthalmoscope which Helmholtz had given him, that the magnitude of this special study became apparent and the way was opened for hitherto undreamed possibilities in ocular medicine and surgery. In otology the wildest vagaries and most impossible theories were taught and accepted until the ear was individualized in anatomy and its normal functions and abnormal processes investigated and studied; and Sir William Wilde, the father of the disciple of astheticism, has by his labors in aural surgery alone, honored Ireland, as Von Troelsch has Germany and Politzer, Austria; and in odontology and oral surgery I need say nothing to you of the honest conscientious work that has made America the dental school of the world, and has given to dentistry its rightful position among the specialties of surgery.

But while we may recognize and can scarcely overestimate the value of specialized labor, we must also recognize a dangerous tendency to which it may give rise.

There is a diseased condition of the retina in which, while the central vision is unimpaired, or even by contrast rendered more acute, the outer field, gradually, perhaps imperceptibly, but none the less surely, is narrowed, until the victim, whose eyes resemble those of other men, is enabled only to see directly before him;—all else is veiled in impenetrable darkness. By a concentration of the powers of intellect upon a single object throughout the months and years, there is danger that the mental focus so maintained may not readily be relaxed again; and the elaboration of the one idea may be to the exclusion of collateral truths. The blind men who for the first time were brought in the presence of an elephant formed their ideas of the creature from the part of his body which they happened to touch The one found his fingers reaching out over the great broad sides of the huge beast, and said at once that an elephant was like a wall. The second grasped the leg, and he thought it more resembled a tree. The third, in whose hands was the writhing, twisting trunk, was sure that an elephant was exactly like a snake. In order that we may comprehend truth in its widest and fullest bearings we must look upon it from all standpoints. We must not consider it in its own totality simply, no matter however carefully our scrutiny of it may be made; but we must understand as well its relative place in our cosmogony. Truth itself is indeed invariable; but even truth may present a veritable phantasmagoria as the medica are changed through which we view it.

Of all mankind, therefore, among none is breadth of thought and comprehensiveness of view more imperative than with the specialist, let his work be what it may. He must synthesize as well as analyze; and the results of his individual research are actually a basis merely upon which general axioms may be grounded. This leads me to the subject about which I wish to speak very briefly to-night, the relations existing between dentistry and otopathy.

The importance of this subject and the interest with which it is invested are at once apparent when we recall the intimacy of the connection between the tissues of the ear and those of the teeth. You will permit me, perhaps, to recall briefly to your memory the anatomy involved. The auricle, you will recollect, is continuous with the external auditory canal. This, with a slight curve in its course, extends to the middle ear, where it is terminated abruptly by the oblique drum-head, or tympanic membrane. The ossiculæ, the three smallest bones in the human body, extend through the tympanum, or drum, forming a chain, one of them being attached to the drum-head, the others extending to the inner ear. This portion of the auditory apparatus consists of three portions—the vestibule, the cochlea and the labyrinth. Of these I shall not speak in detail. Suffice it to say, that within this, the most internal ear, are the nervous structures by means of which the sensation of sound is carried to the brain. From the lower and anterior portion of the tympanum a tube extends to the pharynx, and its mucous membrane is directly continuous with that with which the fauces are lined. It is still a mooted question whether these tubes are normally open or not, but in any event, the atmospheric air passes through them during the act of swallowing. It is to the nervous structures, however, and especially to those of the sympathetic system, by which all of these tissue are connected, that I wish particularly to direct your attention.

The ganglia, as you know, serve as storehouses, or perhaps rather as way stations, at which transfers of nervous energy may occur, and connections be made between unlike structures. Through the medium of these ganglia the relations between the teeth and the ear are made the more intimate. The outer ear, including the auricle and external aural canal, are largely under the influence, it will be remembered, of the auriculo-temporal nerve; and this latter has a small branch passing directly to the otic gangloin. Within the tympanum communications occur between filiments of the glosso-pharyngeal,—the carotid plexus,—the facial, and the otic gangloin, and this same otic gangloin communicates directly with the inferior maxillary from which the dental nerves derive their origin. The chorda tympani encased in mucous membrane passes through the tympanic cavity after which it unites with the gustatory branch of the lingual to supply the tongue, and is therefore, as Cooper has said, "in propinquity with the throat opening of the ear—with the brain opening, as we may call the meatus auditorious internus—as well as with the mastoid openings." It will be remembered, furthermore, that all of the non-striated muscular tissue—including the vascular coatings—is under the influence of the sympathetic nervous system; and when, in addition to all of this, we remember the universal distribution of the mucous tissue in lining the mouth, pharnyx, Eustachian tubes and tympanum, the readiness with which a disturbance in one of these tissues may be reflected upon any one of the others is very evident.

I have given these tedious details in order that some of the disturbances which the eruption of the teeth excite may be the more readily understood.

The sensitive infantile organism is more easily affected by any perversion of the nerve function than is that of adult life, and the change is the more rapid from a physiological to a pathological condition, and Von Trœlsch has definitely pointed out a fact that has since been frequently observed by the physician, that the eruption of each deciduous tooth is often accompanied by an inflammation of the middle ear of the child, an inflammation that frequently results in rupture of the drum-head with purulent discharge. In weakly or badly nourished children, the integrity of the membrane is not readily restored, and a chronic otorrhœa, with consequent deafness, may be the ultimate result. Mr. Cooper, to whose authority reference has already been made, believes that the eclampsia of infants may be often directly traceable to the middle ear as the point of origin, and particularized a case under his own observation in which spasms inaugurated the cutting of each tooth except *when a discharge took place from the ear of the child.*

Very little attention had been given to the study of the teeth as an etiological element in aural disease until Mr. Kempton, an English dental surgeon, urged its importance in a paper entitled "Sympathetic Nervous Affections connected with the Teeth," In this paper the writer demonstrated that the teeth may often be the foci of remote and apparently disconnected difficulties. In Liston's "Elements of Surgery" the following paragraph, inspired by Mr. Kempton's researches, has so direct a bearing upon this subject that I may be permitted to quote it entire. "From the presence of carious teeth," says Mr. Liston, "or decayed portions of teeth, many evils, both local and general, ensue, besides inflammation and abscesses. They are frequently the cause—and the sole cause—of violent and continued headache; of glandular swellings in the neck, terminating in, or combined with, abscesses; of enlargement and inflammation of the tonsils, either acute or chronic; of ulcerations o the tongue and lips—often assuming a malignant action from continued irritation; of painful feelings in the face; tic douloureux; pains in the tongue, jaws, etc.; of disordered stomach from affections of the nerves, or from imperfect mastication; of constitutional irritation, which may give rise to serious diseases." It is very evident that none of these conditions can be relieved or are amenable to treatment, except the irritant or exciting cause be first removed. Mr. Cooper goes so far as to express the belief that by no means an insignificant proportion of the cases of tonsillary hypertrophy, with the consequent, or at least accompanying, deafness, are the direct result of dental caries.

Another very important complication to which I should like to refer as frequently sympathetically involving the ears, is the difficult eruption, the misplacement, or the impaction of the wisdom teeth.

It will be remembered that in the superior maxillary the germs are placed high in the tuberosities of the bone, while those of the lower jaw are imbedded in the cancellous structure at the base of the coronoid process. At intervals of seven years—beginning between the seventh and eighth year of life—the permanent molars usually make their appearance. The first permanent molar—when it is developed—is found between the second temporary molar and the base of the coronoid process. As the temporary molars are replaced by the bicuspids, it is only by the lengthening of the body of the jaw, that room is made for the second permanent molar and the wisdom tooth. These changes which the jaw undergoes are very graphically pictured in the plates of Grey. It will also be seen by these plates that the angle of the ramus is altered in adult life. In a majority of cases these changes do actually take place. As Mr. Kempton has shown, however, in the admirable pamphlet to which I have referred, there are instances in which more or less disturbance is occasioned by "a disproportion in the rate of growth between the tooth and the jaw, or even from an arrest in the growth of the latter—while the former continues in its development. The consequence is that the tooth cannot extricate itself from the jaw for want of space in the horizontal ramus." It occasionally happens, moreover, as all dentists know, that an actual arrest may occur in the evolution of the wisdom tooth for inexplicable reasons. In the practice of Mr. Cooper a case is cited of a lady, forty-three years of age, in whom three of the wisdom teeth have never come forward. Two of these are in the upper and one in the lower jaw. Two of them are plainly visible and evidently but little misplaced. The left lower tooth was so firmly impacted that it resisted all efforts at removal. The left upper tooth, however, was much out of position and was the direct cause of a large cyst that had formed in the bone.

In another case, that had been under the care of Mr. Bryant and Mr. Weiss, a man forty-three years of age had suffered with an abscess between the cheek and gum with fistulous openings bursting both within the mouth and externally upon the cheek, and with this rigidity of the jaw. At the suggestion of one of the surgeons the second molar was removed in order that the wisdom tooth, which had not appeared, and which was believed to be at the root of the mischief, might be examined. A part of the crown which was thus rendered visible showed the third molar to be lying horizontally in the jaw. The subsequent extraction of the misplaced wisdom tooth resulted in the complete relief of the patient. Again, in a case of Mr. Kempton's, and quoted by Liston, a young man had suffered with neuralgic pains in the ear for three years, with occasional fœtid discharge. The pain seemed to be directed to the last molar which, on examination, was found to be defective and was removed. The aural discharge immediately ceased and the otalgia never reappeared. An exceedingly instructive case is reported in Holme's System of Surgery, in which a lady had been a sufferer for twenty-one years with what had been considered a most unmanageable neuralgia. Together with nervous prostration, was loss of hearing, partial paralysis of the muscles of the eye, and impaired vision. The wisdom tooth was absent, and the periostitis of the second molar indicated the probable impaction of the third. An examination discovered the latter to be lying horizontally in the jaw. The second molar was extracted with immediate relief of all the suffering; a month later hearing and vision had so greatly improved that there was every probability of a complete re-establishment of all the normal conditions. In this case twenty years of suffering, and that taken from the middle of life, with a consequent depression of the vital powers for the remainder, was the result of a negligent diagnosis!

Another possible cause of disease of the ear I would refer to very guardedly. My observations have not been of a sufficiently definite character to directly charge red rubber plates and amalgam fillings as exercising a bad influence upon the integrity of the middle ear—but for a long time I have looked upon them with increasing suspicion. The very slow progress and insidious development of a mercurial aural catarrh is so closely allied to that arising from other causes that the differential diagnosis is made with the greatest difficulty. But I am led to believe that in patients easily affected by mercury these are not wholly harmless. A gentleman now under my care, who is suffering from chronic inflammation of the middle ears had worn a red plate for ten years. During that time he suffered almost constantly with canker sores on the lips and mouth. Some time since I suggested the advisability of replacing the red with a black plate. The mouth quickly recovered and none of the sores have appeared since. As I have said it is difficult to determine a matter of this kind. I have observed, however, that among the large number of cases of chronic inflammation of the middle ear that come under my observation annually, a very considerable proportion of them are found in patients wearing red plates; and where it has been possible I have advised the substitution of some material in which mercury is not an ingredient. Of badly fitting plates I think we may speak in more decided terms. There seems to be little doubt that the irritation to which they may give rise may be carried to the throat and thence to the ears. Of the occasional ill effects of amalgam fillings, too, there appears to be little question, for every dentist will recall instances in which the resulting neuralgia has compelled the removal of the amalgam and the substitution of gold. Sufficient data have not yet been accumulated to allow us to formulate a direct charge against the amalgam as an agent in the production of deafness; but from circumstantial evidence we are warranted, at least, in looking upon it with gravest suspicions.

A few words in relation to the teeth as sound conductors, and I will have finished.

It is a well known fact in physics, that on the relative degrees of density and elasticity of medica depend their value as sound conductors. Dry hard wood carries the sound-waves much more readily than does our atmospheric air. Boys will frequently amuse themselves by tapping upon the end of logs forty or fifty feet long—the sound being distinctly audible to the ear of the playmate at the other end, while in the atmosphere no sound can be heard. Nature has not ignored this fact in the function of audition; and the bones of the head serve in no small degree as aids in hearing. In catarrhal deafness, the auditory nerve being never unimpaired, a tuning fork that is quite inaudable when held in proximity to the ear, is distinctly heard if the handle be placed between the teeth; and a watch tick which cannot be heard when held near the ear is at once distinguished if the watch be held against the skull. This fact has been recognized in that large class of auditory aids which have been designated as osteophones, chiefest of which are the audiphone and dentiphone. These consist of some variety of hard rubber fan, the upper edge of which is pressed against the teeth. The sound-waves striking the surface of the galvanized rubber excite vibrations within its substance. These are carried through the teeth and bones of the skull to the ultimate nervous filiments. Any of these instruments are rarely of much practical benefit, as the force of the sound is so much reduced by its indirect reception and transmission before reaching its ultimate destination. A simple rod of dry wood held between the teeth of the speaker and the one spoken to serves a much better purpose. Indeed, a homely appliance of this kind served for many years as the only communication between Beethoven and his instrument, and enabled him again to hear the tones which his genius had evolved long after his ears had been shut to other sounds. But I must cease. I had hoped to show conversely the manner in which certain morbid processes within the ear might be reflected upon the teeth; how waxy accumulations or parasitic growth within the ear canal, or aural neuralgias, might be erroneously diagnosticated or attributed to dental influences; but I fear that I already exceeded the limits of time allotted to me in the have invitation of your committee. I have by no means attempted to deal elaborately with any of the subjects upon which I have touched. My object in these remarks was less to demonstrate the relation which one form of disease may exercise upon another, or the influence which the morbid condition of one organ or tissue may have upon another, than to urge the imperative necessity of studying pathology in *all* of its manifold relations, and of looking upon disease in such a broad and comprehensive way that by the political economy of co-operation the workers in all the special branches may unite in the evolution of a *new* pathology, the basis of which is universal truth. When this shall be done, specialism will have accomplished a far higher mission than the development of manual dexterity simply, for it will have woven the threads by which disassociated facts shall be drawn together, apparently antagonistic truths harmonized, and scattered varieties—now perhaps unrecognized—united in the establishment of a far-reaching and comprehensive pathology.—*Dental Advertiser.*

